# The introduction and current status of the multidimensional model of pain neurobiology

**DOI:** 10.3389/fpain.2023.1161877

**Published:** 2023-04-20

**Authors:** Kenneth L. Casey

**Affiliations:** Professor Emeritus Neurology Molecular and Integrative Physiology, The University of Michigan, Ann Arbor, MI, United States

**Keywords:** pain theory, conceptual models, affect, cognition, sensory discrimination, pain modulation, pain neurobiology

## Abstract

Conceptual models are useful because they guide our practical actions related to whatever is represented by the model; this includes research that reveals the limitations of these actions and the potential for their improvement. These statements apply to many aspects of daily life and especially to pain as a challenge for both clinical practice specifically and neurobiology generally. In the first half of the 20th century, our conceptual model of pain, to the extent that it existed at all, was based on evidence supporting the proposition that pain emerged from activity within a very spatially limited set of central nervous system (CNS) structures located within the cerebral cortex and it's oligosynaptic connections with the thalamus. This CNS activity was strongly associated with the activation of physiologically distinct and specialized somatovisceral afferent fibers. All, or nearly all, aspects of the pain experience were thought to arise from, and be modified by, changes in that localized CNS activity. There was no compelling and widely accepted reason to consider an alternative model. However, neurophysiological, neuroanatomical, behavioral, and clinical evidence emerging in the late mid-20th century prompted a reconsideration of the prevailing model of pain neurobiology. Based on this new evidence and the perceived limitations of the prevailing model, pain could then be reasonably conceived as a multidimensional experience arising from the conjoint activation of physiologically and anatomically distinct but interacting CNS structures each separately mediating sensory discriminative, affective, and cognitive aspects of pain. This brief historical review describes the intellectual climate at the time this multidimensional model was proposed, the dispositions for resisting or accepting it, and concludes with a comment on the current status of the model as a fusion of distributed activations that create a unified perception of pain.

## Introduction

Why review the history of research into the neurobiological mechanisms of pain? How did we arrive at our current level of understanding? For some readers, it may simply be an interesting and informative story. For others, such a review may have more practical implications. After all, physical pain, the perception of somatic or visceral injury, is among the most compelling experiences of almost all presumably sentient animals and, because it is threatening and unpleasant, we wish to know how to avoid, attenuate, or eliminate it. Learning about how we have arrived at contemporary concepts of pain mechanisms can reveal how and why various concepts have evolved as new information appears. Knowing this history may improve our ongoing search for improvements in treatment and, more broadly, in understanding the mechanistic function of nervous systems generally.

From a practical clinical perspective, we may be able to avoid historical missteps and blind alleys that lead to harmful medical practices and serious misunderstandings of neurological functions. For example, in a previous contribution to this series on the history of pain research, Basbaum (1) has presented examples of how attempts to eliminate pain selectively by surgically interrupting the anteriolateral fasciculus in the anterolateral quadrant of the spinal cord (the spinothalamic tract) may lead, over variable time periods, to a variety of serious and often painful postoperative complications including a return of pain and the development of new pain. In a recent literature review of 45 reports of the results of open and percutaneous anterolateral cordotomy, Javed et al. (2) conclude that: “Today, spinal cord ablation is almost exclusively used for refractory cancer pain and in the palliative care setting…” (pg. 291). This conclusion is reached in large part because of the temporally limited clinical benefit of this procedure and its attendant complications but also because of our improved understanding of pain neurobiology and the development of more effective, less invasive methods of managing acute and chronic pain.

The medical rationale for a surgical spinothalamic tractotomy and similar ablative procedures has been based on the concept of a “pain pathway” that activates a supraspinal target that specifically and selectively creates pain. That rationale was based, in turn, on the broader concept of *functional localization* that has been undergoing revision throughout the late 20th century to the present. Rather than becoming mired in a consideration of what is meant exactly by functional localization, and in accord with the focus of this Frontiers series on pivotal moments in pain research, I will consider here these broader concepts only as applied to pain.

As many of our readers know, pain is currently defined by the International Association for the Study of Pain (IASP)) as: “…*an unpleasant sensory and emotional experience associated with, or resembling that associated with, actual or potential tissue damage.*” (3). I will review briefly the scientific background prevailing at the time this current concept of pain as a multidimensional sensory and emotional experience began to emerge. I will often use the word “affect” or “affective” in reference to the phrases “emotional experience” in the IASP definition or “motivational-affective”, which is sometimes used to refer to this component of pain. Some of what is reviewed here has been discussed elsewhere (4).

## The skin senses symposium

From an historical perspective, it is instructive to review The Proceedings of the First International Symposium on the Skin Senses (hereafter the SS Symposium), held at The Florida State University, Tallahassee, Florida, U.S.A. in March of 1966 (5). This Symposium was attended by 45 international investigators who contributed 29 chapters on various aspects of somatic and visceral sensation. The Society for Neuroscience and the International Association for the Study of Pain did not exist. The various subdisciplines of Neuroscience (anatomy, physiology, biochemistry, psychology, and related clinical specialties) were represented by separate organizations with their own publications. This published volume, then, approximately reflects the direction and emphasis, at that time, of research on somesthesis generally, including pain.

In overview, most of the research presented and discussed in this publication consisted of elegant studies of the anatomical and physiological properties of somatic and visceral afferent nerve fibers and the receptors they innervate. At the time, there was substantial evidence that information about potential or actual tissue damage, and therefore pain, required the activation of afferent fibers with high electrical and mechanical or thermal thresholds and that these fibers were finely myelinated or unmyelinated (6–11). It is important to recall, however, that the discovery of single nociceptive afferent fibers, as defined by their selective or differential response to noxious thermal and mechanical stimulation of mammalian skin, was not published until a year after the SS Symposium convened (12).

At the time of this Symposium there was also strong evidence that the afferent activity entering the spinal cord or brainstem was strongly modulated by presynaptic and postsynaptic interactions among active afferent fibers and by activity descending from supraspinal and suprabulbar sources (13–16). The neuroscientific and clinical evidence then available led to the publication of new hypotheses about somesthetic mechanisms generally (17) and then to the gate control (GC) hypothesis of pain (18). The historical background of these seminal conceptual contributions has been reviewed previously (19, 20). For the present purpose, it is necessary to note that much of our current information was not available at the time of the SS Symposium and that detailed and definitive studies of the neurophysiology of spinothalamic dorsal horn neurons would not be available for several years later (21).

The newly introduced GC hypothesis presented clinical and experimental evidence that pain did not depend only on the activation of nociceptive-specific afferent fibers but could be created and modulated by interactions among physiologically heterogeneous afferent fibers and supraspinal and suprabulbar activity. The GC hypothesis and its neurobiological foundation had received some discussion in the literature (14, 22) but was not the focus of this Symposium probably because of its status as a very recent and intellectually novel, even controversial, proposal. Nonetheless, in presenting diagrammatic summaries of the organization of sensory processing in the spinal dorsal horn, Patrick Wall referenced the neurophysiological and anatomical foundations of the GC hypothesis and the earlier speculation on cutaneous sensory mechanisms while commenting that the discriminative capacity of the central nervous system (CNS) does not “…mean that every discriminable event is recognized in the CNS by the appearance of activity in a specific set of fibers which remain silent in the presence of all other events.” (5, Ch. 26, pg. 512). There was, then, a clear challenge to what has been called “specificity theory”, especially with reference to pain mechanisms. The open discussion sections in the Symposium publication reflect the emerging and ongoing debate about that issue.

However, although there was some discussion about the sensory consequences of afferent fiber activity, in all the presentations in which pain was studied explicitly, an affective component of pain was not considered or discussed as a primary critical variable; this is possibly because they were conducted in laboratory settings in which this aspect of pain, if relevant at all, was presumed to be controlled and therefore not a determinant of the experimental result. The issue of determining sensory “quality”, presumably related at least somewhat to the affective content of sensory experience, was briefly mentioned in Nafe's introductory chapter (5, Ch.1 pg. 5*)* but was set aside to focus on the sense of innocuous pressure discrimination. Gagge and Stevens (5, Ch.16 pg. 345) showed that a psychophysical measure of ambient, whole body *thermal comfort* could be used to quantify differential changes in comfort associated with warming and cooling within a closed chamber. However, these careful experiments were all conducted well outside the range of noxious intensities and there was no discussion of the neurophysiological or anatomical connection among thermal discriminative and affective mechanisms. The possibility of an affective relationship between tactile spatial and pressure intensity discrimination was not considered in a paper on the differential tactile sensitivity among body areas (5, Ch.10 pg.195); nor was the issue of affect raised in Gibson's studies of electrical stimulation of touch and pain threshold (5, Ch.11 pg. 223). However, that study focused on developing painless electrical stimuli as a means of communication. An affective component of pain was not considered in the experiments of Hardy et al. (5, Ch. 21 pg. 444) on heat pain threshold and suprathreshold intensity following immersion of the hand in a water bath. Even in Patrick Wall's detailed discussion of the anatomy and electrophysiology of the dorsal horn in the context of GC theory, pain was mentioned only once (5. Ch.26 pg. 513) and without any reference to an affective component of pain.

The concept of a “pain pathway” was strongly reinforced by the recent identification of a putative target for this pathway within the ventroposterolateral thalamus where neurons responding exclusively to noxious somatic stimuli could be found (23–25; however see also 26). These findings were consistent with the clinical observation that pathological lesions within the ventrolateral region of the thalamus, although sometimes producing the poorly understood painful “thalamic (central pain) syndrome” often severely impaired pain as well as tactile and kinesthetic sensations (4, 27, 28). The cerebral cortical target of these thalamic neurons was uncertain but anatomical and clinical evidence available at the time suggested that neurons within the primary somatosensory parietal (S1) cortex could mediate the full experience of pain. For example, in the late 1940's and early 1950's, 18 World War (WW) 2 and some WW 1 soldiers with bullet wounds of the skull appeared to have suffered damage primarily or even exclusively the S1 cortex (29, 30). Four of these patients, when clinically examined months or years following the injury, had reduced or even absent pain and temperature sensation, usually accompanied by impaired tactile discrimination, in parts of a hand, arm, or leg. The neurophysiological basis for these results remains largely unexplained today although direct or retrograde subcortical thalamic or brain stem damage from these high-velocity impacts could not be ruled out (31). Considered together, these findings provided evidence for the termination of a “pain pathway” in the somatosensory (S1) cerebral cortex. Thus, there were several reasons why any interaction among sensory and affective neural mechanisms related to pain was not a salient consideration.

At the time of the SS Symposium, there was considerable evidence that multiple interconnected structures within the mesial and temporal forebrain with oligosynaptic connections to the hypothalamus, mediated aversive behavioral and autonomic responses to noxious somatic or visceral stimulation. Based largely on the neuroanatomical evidence marshalled by Papez (32), MacLean (33, 34) proposed that these structures comprised a “limbic system” that encircled the upper brainstem and mediated a wide range of emotional experiences. Although the limbic system formulation has received ongoing criticisms based largely on defining precisely its functional composition (35), its foundational basis was supported by early experiments (36, 37) showing that noxious somatic stimuli consistently evoked rage-like and other aversive behaviors in decerebrated mammals if the hypothalamus and rostral brainstem remained intact. Woodworth and Sherrington speculated that these “pseudaffective” responses would likely have been painful had the forebrain remained intact (36). Subsequent studies showed that the mammalian mesial upper midbrain, hypothalamus, and medial thalamus were anatomically strongly connected to limbic forebrain structures (38). Aversive and appetitive behaviors could be evoked separately by focal electrical stimulation within limbic system structures such as the hypothalamus, septal nuclei, amygdala, cingulate cortex and hippocampus (39). Clinical experience confirmed that pathological or surgical lesions within cortical and subcortical limbic system structures produced a selective or predominantly affective blunting of the pain normally produced by painful disease or during neurological examination (for review, see 40, Ch.9).

Thus, there was substantial but incomplete evidence available at the convening of this Symposium to support combining the affective and discriminative mechanisms into at least a working or conceptual definition of pain sufficient to guide future discussion, research, and clinical practice. Why was this opportunity set aside or ignored at the time? And what were the circumstances that then prompted the introduction of an addition to the recently proposed and debated GC model of pain? The answers to these questions are conjectures only. Nonetheless, because I participated in this Symposium and have committed to comment on these issues, I will offer some thoughts.

A major reason for indifference or resistance to combining affective and somatovisceral sensory processing into a neural mechanism for pain was simply that it was widely considered unnecessary and probably mechanistically incorrect. Pain is normally easily recognized by all humans as a *unified* sensory experience localized in space and time, varying in intensity and descriptive quality (sharp, dull, burning, aching, etc.) but clearly and reliably distinguishable from all other experiences, including other somatovisceral sensations. On introspective grounds alone it is reasonable to categorize pain as a unique “primary” or “fundamental” sensation and therefore created, like other sensations, by a separate, anatomically and physiologically distinct neuronal system. This model, supported by the evidence then at hand, considered the affective experience to be a consequence of pain, rather than a mechanistically inseparable component of the experience. The affective component could then be considered the “reaction to pain”, presumed to be imperceptibly delayed, and placed into the category of a cognitive evaluation mediated by an independent, separate neuronal system. The same mechanistic formulation could be applied to all other sensations that might have an affective component also although only under specific circumstances and less reliably. There may also have been some underlying resistance to the then nascent concept of distributed neuronal systems mediating relatively “simple” sensorimotor functions.

**Figure 1:**
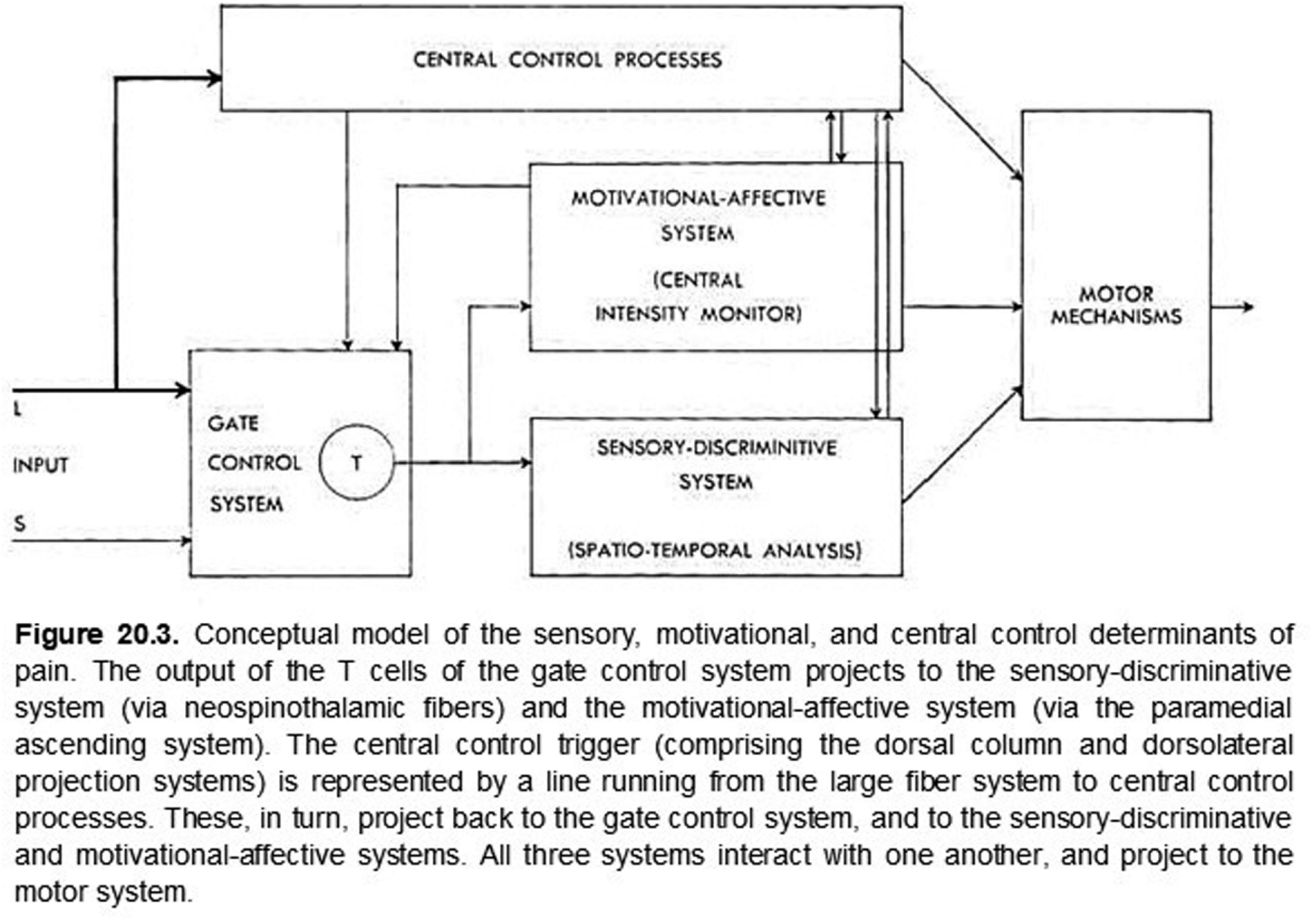
ORIGINAL FIGURE 20.3 (WITH LEGEND)

**Figure 2:**
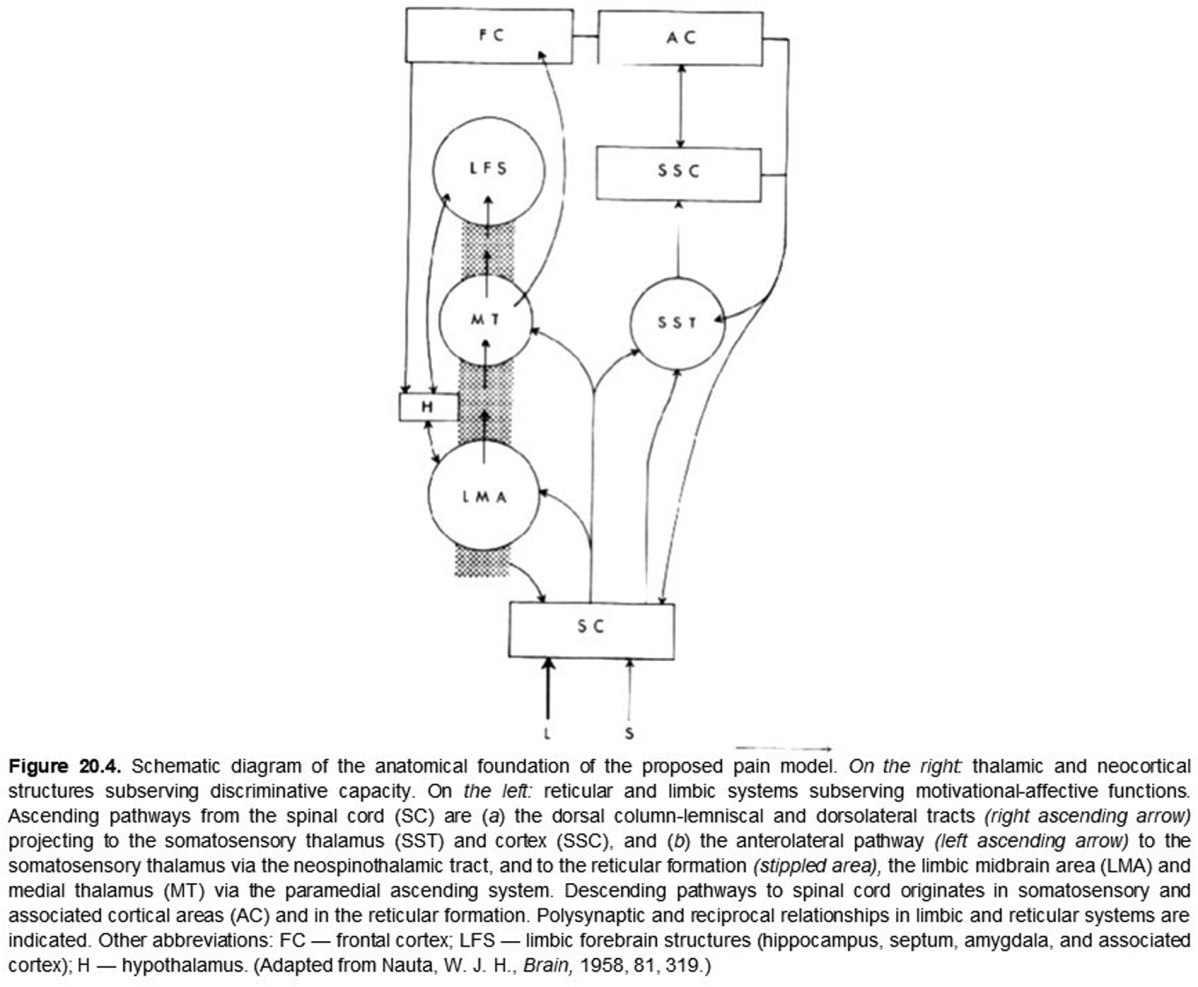
ORIGINAL FIGURE 20.4 (WITH LEGEND)

So what brought discriminative and affective mechanisms together for pain? In the late 1950's and early 60's, there was a near coincidence of neuroanatomical, neurophysiological, and related publications that focused attention on the somatovisceral senses generally and pain in particular. Neuroanatomical studies had revealed oligosynaptic interconnections of subcortical structures, including the anterolateral spinal cord (spinothalamic tract), the brainstem reticular formation, midbrain central gray matter, hypothalamus, ventrolateral posterior and medial-intralaminar thalamus with limbic forebrain structures (38, 41). Neurophysiological investigations found that neurons responding either differentially or exclusively to electrical stimulation of the spinothalamic tract and to presumably noxious somatic stimuli were found not only in the ventrolateral posterior thalamus but also in the medial-intralaminar thalamus (25). The nociceptive responses of some of these cells had just been shown to vary with the level of arousal in awake monkeys (26). Finally, as noted above, Melzack and Wall (17) had recently published their challenge to “specificity theory” as an organizing principle of somesthesis generally. These publications, together with the previously referenced intracerebral stimulation behavioral studies (39), established a background incentive for combining somatovisceral discriminative and affective neuronal mechanisms into a preliminary working model of pain neurobiology (see Figures 1, 2).

As additional background, it should be noted that, before the Symposium was organized, Ronald Melzack recognized that the GC theory did not address adequately a mechanism for the affective dimension of pain. I was informed that Patrick Wall was much less concerned about that issue. I had developed a neuronal population perspective of somesthetic mechanisms under the research mentorship of Drs. Arnold Towe and Suhayl Jabbur at the University of Washington (Seattle). I had recently completed a Selective Service tour of duty in Dr. Paul Maclean's Section on Limbic Integration and Behavior at the National Institutes of Health where I had the opportunity to interact with Drs. Nauta and Mehler (1963–4). Those experiences formed the basis for my attraction to the just-published GC theory and the opportunity to work at McGll University with Ron on the conceptual synthesis of somatovisceral and affective mechanisms into the working model of pain suggested by the definition cited above.

## Current Status and a proposal

For numerous reasons, the neurobiology of pain remains a topic of considerable interest, vigorous discussion, and debate (6, 42). Yet, at this stage of our understanding, perhaps we can agree that all sensory experiences are created by the *perceptual fusion* of coactivated, physically distinct but interacting discriminative and affective neuronal mechanisms and that the emerging sensation is determined by the degree to which these mechanisms are coactivated. At the extremes, some sensations may be nearly devoid of affective content because of weak coactivation while others, such as pain, are created and modulated (43) by the complete, or nearly complete, coactivation of these distinct mechanisms, thus rendering their distinction normally imperceptible. In rare clinical cases, it is possible to reveal a nearly complete dissociation of these intrinsic discriminative and affective components of pain (44, 45). As proposed originally (5, Ch. 20; Figures 1, 2), both aspects of pain are modulated by cognitive mechanisms such as expectation, fear, and mnemonic processes (43) that may not be temporally locked to an afferent barrage.

The coactivation issue appears to be the fundamental problem addressed so thoroughly by Price (46) and specifically by Fields (47) in his scholarly argument for introducing a word, *algosity*, (italics mine) to express the imperceptibility of this perceptual fusion in the case of pain. Both of these scholars expressed clearly the problem of distinguishing among discriminative, intrinsically affective, and cognitively modified experiences especially in the case of pain. As they suggested, this issue was not clearly examined adequately in the original presentation of this multidimensional model of pain.

What, then, determines the critically important degree of coactivation in this model? The simple, honest answer is that this is not known. An attempt at a comprehensive answer to this question would certainly invoke numerous interacting factors and would require a discourse beyond both the purpose of this communication and the competence of its author. It will suffice here to refer briefly only to the broad range of neural mechanisms subsumed under the category “cognitive” and instead emphasize, specifically for pain, the well-known critical importance of the composition of the somatovisceral afferent volley. It is clear that the tissue receptors innervated by unmyelinated (C) and lightly myelinated (A delta) fibers normally have preferential access to affective neural systems, thus enhancing the likelihood of their coactivation with discriminative mechanisms. How this differential coactivation is accomplished by differences in the composition and origin (9, 48) of an afferent input is also unknown and remains an area of active research. Meanwhile, as a practical clinical matter, it is reasonable to expect that a variety of neurological disorders, both central and peripheral, can alter the degree of this coactivation, creating abnormal sensations with the altered, usually exaggerated but, rarely, attenuated (45), affective content typical of neuropathic pain. Understanding the mechanisms underlying these clinical presentations should help guide us toward a wider range of less harmful approaches to diagnosis and therapy.

I am tempted here to create a Venn diagram to depict the coactivation model suggested above but perhaps we have enough diagrams about this subject for now so I will defer and hope my words will suffice, leaving any depictions for others to create.

## Data Availability

The original contributions presented in the study are included in the article/Supplementary Material, further inquiries can be directed to the corresponding author/s.
